# Progress of Surgery

**Published:** 1901-03-02

**Authors:** 


### Progress of Surgery.

### HERNIA.

**Intra-abdominal Omental Torsion.**—This subject has recently received special attention, and a very complete paper concerning it has appeared by J. Wiener, jun.[1] The condition was first recorded in 1882 as occurring in an inguinal hernia, and in 1896 a case was recorded in which an omental hernia had undergone retrograde incarceration, *i.e.*, the omentum was sling-like, the terminal portion having re-entered the abdomen through the stricture and become gangrenous. A little later such a condition was described in association with torsion of the omental pedicle. The few recorded cases have usually been in association with hernia. It occurs more often in males than females, and commonly after middle age. In many cases the exciting cause has been forcible reduction of the hernia.

**Umbilical Hernia.**—In a case with very pendulous abdomen "retrenchment" of the abdominal wall was performed at the time of the cure of the hernia by Jas. B. Bullit.[2] A transverse incision about 6 inches long was made 2 inches above the umbilicus and the sac and hernial ring exposed. The omentum and colon were returned, the sac removed, and the ring closed. Several pounds of skin and fat were next resected from the abdominal wall, and sutures applied in tiers. The result proved quite satisfactory.

**Gangrenous Hernia.**—Dr. C. L. Gibson, of New York, has analysed 1,000 cases of acute intestinal obstruction and gangrenous hernia.[3] He finds that more femoral herniæ are found gangrenous than in the inguinal variety. Perhaps this is from the reasons that they give rise at first to few signs to excite observation, that mortification is rapid when strangulation has once started, and that reduction is difficult. Gangrenous umbilical hernia is particularly severe in its consequences. Indeed, a very considerable portion of the intestine may require resection, amounting perhaps to several metres. Of the greatest value, however, are the statistics given as to the results of treatment for gangrenous hernia. "The figures demonstrate a marked superiority of resection and primary enterorrhaphy over artificial anus with or without resection of the gangrenous intestine." Of the methods of union Gibson speaks very favourably of the Murphy button. Its use in 63 cases gave a mortality of less than 25 per cent., a truly wonderful result under the circumstances. Early operation is of paramount importance. Nine cases resected on the first day of obstruction all recovered. Resection with artificial anus has a mortality of 50 per cent. It is perhaps difficult to understand why the mortality should be so much greater here. But it must be remembered that the superadded mortality of the closure of the artificial anus is great.

It is curious that the rate of mortality in union by suture and by mechanical aids is about the same in each. But the impression conveyed to the writer is that mechanical aids (the Murphy button being taken as the best) give a lower mortality by some 9 per cent., and it is certainly better for surgeons inexperienced in intestinal suture, and at the same time extends the field of the operation of lower mortality, viz. resection. Considering the possibility of removing very large portions of gut, it is a noteworthy fact that 12 cases died from insufficient removal of gangrenous bowel. A "generous margin" of healthy bowel should therefore be removed, particularly as gangrene is more extensive within the bowel than without it.

Another important point is that 10 per cent. of the mortality of resection cases were from mesenteric hæmorrhage. The mortality of artificial anus in obstruction cases is very high—viz., nearly 80 per cent. for intestinal obstruction, and over 50 per cent. for hernia. Of the causes of death in 1,000 cases a quarter were due to shock or to peritonitis. No less than 14 cases died on the table. One of the most important measures against shock is rapidity of operation. The remaining details in which improvement can be hoped for are in the efficacy of the search for the cause of obstruction, and a thorough appreciation of the best measure to adopt at the moment. It is important that all forms of suture likely to be required should be in readiness to avoid loss of time. Evisceration may save time but may cause much shock.

Gibson states that the prognosis of operation for intestinal obstruction depends on the following points, in order:—(*a*) The duration of obstruction, (*b*) the extent and severity of the changes in the intestine, (*c*) the nature and character of the obstruction, (*d*) the judgment and skill of the surgeon, (*e*) the patient's general condition. Altogether Gibson's paper is a model of classification and deduction, of the greatest value to the abdominal surgeon.

**Inguinal Hernia.**—A useful paper has appeared from the pen of Mr. Hamilton Russell, of Melbourne,[4] upon the question whether operation is indicated on all inguinal herniæ except those in young infants. He contends that while the funicular process of peritoneum may undergo obliteration during infancy, it never does so in childhood or later, even if a truss be worn. Mr. Russell combats the generally-received opinion that a hernia in a child can almost always be cured by a skilfully-applied truss, that operation is therefore rarely called for in children, and that the pressure of a truss brings about obliteration of the sac. Moreover, he disbelieves in an acquired oblique inguinal hernia, which he associated with the existence of a congenital funicular process remaining patent. He states, "the presence of a congenital hernia is the sole cause of inguinal hernia, and that operative removal of the sac is the only treatment that is surgically sound." He absolutely discards Bassini's method for children, and simply relies upon removal of the sac for a cure. He regards an acquired sac as a practical impossibility, for the fibres arching over the internal abdominal ring, by their contraction on every strain, act as a true sphincter so as to prevent such an occurrence. Then, again, he says that hernia should not recur after removal of the sac. Such cases as have been recorded as recurring have, he thinks, been the result of traumatism inflicted at the time of operation.

Seeing that there is no oblique inguinal hernia without a congenital sac, and seeing that (in the writer's opinion) a truss never occludes that sac, then Mr. Russell holds that all oblique inguinal herniæ should be operated on and the sac removed, otherwise a cure can never be pronounced. Care should be exercised not to ligature the sac too high up, lest a portion of the bladder wall be included. If, therefore, these arguments be correct the removal of the sac in the herniæ of childhood should never be followed by an oblique inguinal hernia later on in life. Only experience can teach us the truth of this dictum.

**Anatomical Considerations in Radical Cure.**—An elaborate paper has appeared on this subject by Dr. J. S. Haynes.[5] Besides the natural external inguinal fossa of peritoneum corresponding to the internal abdominal ring, we have the several structures of the cord converging at that spot. The writer differs from Mr. Russell, previously referred to, in believing that an acquired oblique inguinal hernia can exist independently of a funicular process. To reconstruct the inguinal canal he advocates the complete Bassini method, including division of the muscular fibres of the internal oblique. The paper is based upon anatomical considerations and is well illustrated.

**Local Anæsthesia in Radical Cure.**—As some surgeons now advocate this measure in preference to a general anæsthetic, reference may be made to an excellent description of the patient's sensation from the personal experiences of a medical man. "If he wants to know how it would feel, let him give his testicles a good hard squeeze, and imagine the pain lasting from thirty to fifty minutes" (Leo Meyer[6]).

[1] Ann. Surg., Nov. 1900. [2] Ibid. [3] Ibid. [4] Lancet Oct. 20, 1900. [5] Med. Rec., Oct. 13, 1900. [6] Ibid, Dec. 22., 1900.

